# Reversal of Insulin Resistance in Overweight and Obese Subjects by *trans*-Resveratrol and Hesperetin Combination—Link to Dysglycemia, Blood Pressure, Dyslipidemia, and Low-Grade Inflammation

**DOI:** 10.3390/nu13072374

**Published:** 2021-07-11

**Authors:** Naila Rabbani, Mingzhan Xue, Martin O. Weickert, Paul J. Thornalley

**Affiliations:** 1 Department of Basic Medical Science, College of Medicine, QU Health, Qatar University, Doha P.O. Box 2713, Qatar; n.rabbani@qu.edu.qa; 2 Diabetes Research Center, Qatar Biomedical Research Institute, Hamad Bin Khalifa University, Qatar Foundation, Doha P.O. Box 34110, Qatar; mxue@hbku.edu.qa; 3 Endocrinology & Metabolism, Warwickshire Institute for the Study of Diabetes, University Hospitals of Coventry & Warwickshire NHS Trust, Coventry CV2 2DX, UK; Martin.Weickert@uhcw.nhs.uk

**Keywords:** polyphenol, insulin resistance, methylglyoxal, obesity, glyoxalase, low-grade inflammation

## Abstract

The dietary supplement, *trans*-resveratrol and hesperetin combination (tRES-HESP), induces expression of glyoxalase 1, countering the accumulation of reactive dicarbonyl glycating agent, methylglyoxal (MG), in overweight and obese subjects. tRES-HESP produced reversal of insulin resistance, improving dysglycemia and low-grade inflammation in a randomized, double-blind, placebo-controlled crossover study. Herein, we report further analysis of study variables. MG metabolism-related variables correlated with BMI, dysglycemia, vascular inflammation, blood pressure, and dyslipidemia. With tRES-HESP treatment, plasma MG correlated negatively with endothelial independent arterial dilatation (r = −0.48, *p* < 0.05) and negatively with peripheral blood mononuclear cell (PBMC) quinone reductase activity (r = −0.68, *p* < 0.05)—a marker of the activation status of transcription factor Nrf2. For change from baseline of PBMC gene expression with tRES-HESP treatment, Glo1 expression correlated negatively with change in the oral glucose tolerance test area-under-the-curve plasma glucose (ΔAUGg) (r = −0.56, *p* < 0.05) and thioredoxin interacting protein (TXNIP) correlated positively with ΔAUGg (r = 0.59, *p* < 0.05). Tumor necrosis factor-α (TNFα) correlated positively with change in fasting plasma glucose (r = 0.70, *p* < 0.001) and negatively with change in insulin sensitivity (r = −0.68, *p* < 0.01). These correlations were not present with placebo. tRES-HESP decreased low-grade inflammation, characterized by decreased expression of CCL2, COX-2, IL-8, and RAGE. Changes in CCL2, IL-8, and RAGE were intercorrelated and all correlated positively with changes in MLXIP, MAFF, MAFG, NCF1, and FTH1, and negatively with changes in HMOX1 and TKT; changes in IL-8 also correlated positively with change in COX-2. Total urinary excretion of tRES and HESP metabolites were strongly correlated. These findings suggest tRES-HESP counters MG accumulation and protein glycation, decreasing activation of the unfolded protein response and expression of TXNIP and TNFα, producing reversal of insulin resistance. tRES-HESP is suitable for further evaluation for treatment of insulin resistance and related disorders.

## 1. Introduction

Epidemiological studies suggest a diet rich in polyphenols decreases the risk of developing type 2 diabetes mellitus (T2DM) [1]. This has led to the evaluation of polyphenol dietary supplements for improvement of metabolic health and improved prevention of T2DM. *trans*-Resveratrol (tRES), a polyphenolic stilbenoid, improved metabolic health and survival of mice on a high-calorie diet [2]. Similar effects have been difficult to translate clinically with meta-analysis of intervention studies concluding that tRES does not affect glycemic status in overweight and obese human subjects [3]. A barrier to effective clinical translation of expected health benefits of polyphenols is limited clinical potency when given individually [4]. We therefore explored synergistic combination of tRES with other polyphenols and targeted insulin resistance for treatment—the driver of development of T2DM [5].

**Figure 1:**
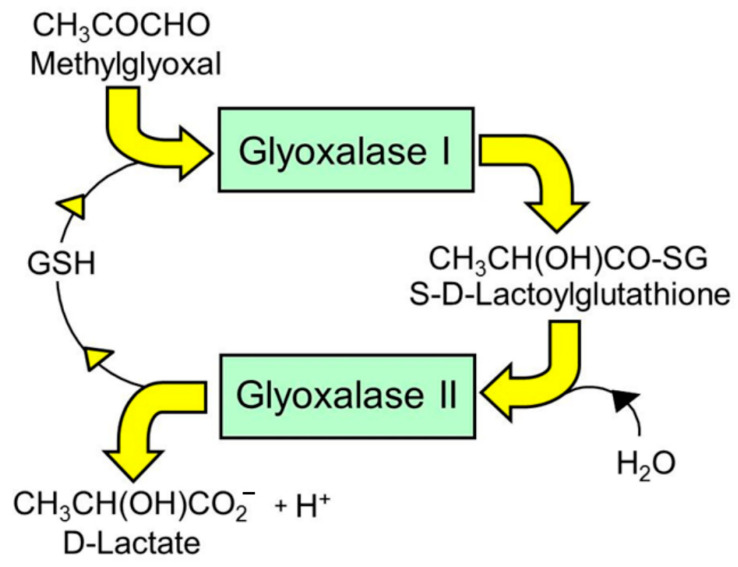
Metabolism of methylglyoxal by the glyoxalase pathway.

Preclinical and clinical studies suggest a role of increased protein glycation by the endogenous reactive dicarbonyl metabolite, methylglyoxal (MG), in the development of insulin resistance and T2DM [6]. MG modifies proteins to form the major advanced glycation endproduct (AGE), arginine-derived hydroimidazolone, MG-H1 [7,8]. MG-modified proteins are misfolded and activate the unfolded protein response (UPR) and downstream inflammatory signaling [9,10]. MG is metabolized by glyoxalase 1 (Glo1), the first enzyme of the glyoxalase pathway which metabolizes MG, to D-lactate [11] (Figure 1). Plasma D-lactate concentration is a surrogate indicator of flux of formation of MG [6,9,12]. Decrease of MG concentration, related protein glycation and alleviation of the UPR-linked inflammation may offer a route to novel insulin sensitizing agents [13]. Inducers of expression of Glo1, Glo1 inducers, may support this by suppressing MG concentration [6]. We developed a pharmacological strategy to induce Glo1 expression, through activation of transcriptional factor Nrf2 and its binding of a functional antioxidant response element (ARE) in the GLO1 gene [14]. In a screen of dietary bioactive compounds that activate Nrf2, we found the synergistic combination of tRES and hesperetin (HESP), tRES-HESP, was the most effective Glo1 inducer [15]. In a randomized, placebo-controlled crossover study in overweight and obese subjects—the Healthy Aging Through Functional Food (HATFF) study—treatment with tRES-HESP reversed insulin resistance, with an improvement of insulin sensitivity in obese subjects comparable to outcomes of metabolic surgery. The placebo had no effect. There were also improvements in dysglycemia and low-grade inflammation [15]. The aim of this study was to explore evidence for association of target pharmacology of Glo1 inducer, tRES-HESP, to clinical variables of insulin resistance, dysglycemia, blood pressure, dyslipidemia, and low-grade inflammation assessed in the HATFF study—including established mediators of insulin resistance, thioredoxin interacting protein (TXNIP), and tumor necrosis factor-α.

Herein, we present further analysis of clinical and biochemical variables of the HATFF study, particularly correlation analysis of glyoxalase pathway, clinical variables and peripheral blood mononuclear cell (PBMC) gene expression. The outcome reveals, for the first time, a link of target pharmacology to insulin resistance, dysglycemia, blood pressure, dyslipidemia, and low-grade inflammation—including key mediators of insulin resistance, TXNIP, and TNFα.

## 2. Materials and Methods

### 2.1. HATFF Study Design and Methods

HATFF is a randomized, double-blind, placebo-controlled crossover study evaluating treatment with tRES-HESP against insulin resistance in healthy overweight and obese subjects. Subject recruitment, including inclusion and exclusion criteria, power analysis, and pharmacological target validation (increase of Glo1 activity and decrease of plasma MG and MG-mediated protein glycation) have been described previously [15]. tRES-HESP was evaluated in 29 subjects with impaired metabolic health; 9 subjects meeting criteria of prediabetes. Twenty participants were highly overweight and obese (BMI ≥ 27.5 kg/m^2^) and 11 were obese (BMI ≥ 30 kg/m^2^). Subjects made 4 study visits for clinical assessments; one visit at the start and end of each treatment period. Treatment was by daily oral capsule before breakfast, containing with tRES-HESP (90 mg tRES, 120 mg HESP) or placebo, for 8 weeks, with 6 weeks washout between crossover treatment periods [15]. The study was approved by the National Research Ethics Service Committee West Midlands—Coventry & Warwickshire, U.K. (project number 13/WM/0368) and registered on the Clinicaltrials.gov (identifier: NCT02095873). The procedures followed were in accordance with institutional guidelines and the Declaration of Helsinki.

The primary endpoint was insulin sensitivity measured by oral glucose insulin sensitivity (OGIS) index in oral glucose tolerance tests (OGTTs) at the start and end of each treatment period. The OGIS index correlates very strongly with reference method assessment of insulin resistance by the hyperinsulinemic glucose clamp method [16]. Secondary endpoints were brachial artery flow-mediated dilatation response, also including brachial artery dilatation response to a sub-therapeutic dose of glyceryl trinitrate (FMD-GTN). Variables characterizing clinical status, glyoxalase pathway-linked metabolism, dysglycemia—including fasting plasma glucose (FPG), area-under-the-curve of the OGTT (AUCg), fasting insulin, and 90 min OGTT plasma insulin (plasma insulin OGTT)—vascular inflammation, blood pressure, and dyslipidemia were recorded and determined, as described [15]. Gene expression in PBMCs was assessed in a custom focussed 50-gene array, including 47 response and 3 reference genes, by the Nanostring method assessing relative mRNA copy number; RNA was extracted and quantified in-house with sample analysis out-sourced to the Nanostring Facility Service, University College, London, UK [15]. All sample analysis was performed with the investigator blinded to the subject and study group origin.

### 2.2. Target Pharmacology of Glyoxalase 1 Inducer

Glo1 activity and mRNA of PBMCs were assayed by spectrophotometric and Nanostring methods, respectively [17,18]. The plasma concentration of MG and plasma protein and urine content of MG-derived AGE, MG-H1, were measured by stable isotopic dilution analysis liquid chromatography-tandem mass spectrometry [19,20]. Plasma D-lactate was assayed by endpoint enzymatic assay by microplate fluorimetry [21]. Total tRES and HESP urinary metabolites were determined by stable isotopic LC-MS/MS after deconjugation of glucuronides and sulfates, as described [15].

### 2.3. Correlation Analysis

For glyoxalase pathway and clinical variables, we assessed the correlation between variables: (i) throughout the study, combining data from all time points and different treatments; and (ii) within treatment periods, tRES-HESP and placebo—exploring correlations of the established hypothesis linking MG metabolism to BMI, dysglycemia, insulin resistance, vascular inflammation, blood pressure, and dyslipidemia. We also assessed correlations of gene expression in PBMCs in treatment periods wherein we explored correlations with statistically significant major changes of gene expression in the tRES-HESP treatment: namely, for monocyte chemoattractant protein-1 (MCP-1, gene CCL2), interleukin-8 (IL-8), cyclo-oxygenase-2—also known as prostaglandin synthase-2 (COX-2, gene PTGS2), and the receptor for advanced glycation endproducts (RAGE, gene AGER). Analysis was made for two study groups: all subjects, and highly overweight and obese subject groups—as previously described [15]. 

### 2.4. Statistical Analysis

Correlation analysis was by the non-parametric Spearman method. For correlations of gene expression in PBMCs in treatment periods without prior established hypothesis, a Bonferroni correction of 47 was applied. Where change in variable was analyzed, this referred to change from the start to the end of the 8-week treatment period. Statistical analysis was performed using IBM SPSS Statistics for Windows, version 24 (IBM Corp., Armonk, NY, USA). 

## 3. Results

### 3.1. Correlation Analysis of Glyoxalase Pathway and Clinical Variables throughout the Study

The outcomes of the HATFF study were found in highly overweight and obese subjects where the following changes were recorded in the tRES-HESP treatment period: target pharmacology—increased in PBMC activity of Glo1 (+27%, *p* < 0.05) and decreased plasma MG concentration (−37%, *p* < 0.05); clinical endpoint-related variables—decreased FPG (−5%, *p* < 0.010), decreased AUCg (−8%, *p* < 0.05) and increased OGIS (+54 mlmin^−1^m^−2^, *p* < 0.05); and other—decreased expression of MCP-1, IL-8, COX-2, and RAGE in PBMCs. The placebo had no effect [15]. tRES-HESP treatment increased urinary excretion of tRES and HESP metabolites by > 2000- and > 100-fold, respectively, compared to the placebo.

Induction of expression and activity of Glo1 with related change in target pharmacology provided the opportunity to explore the association by correlation analysis of glyoxalase system-linked variables with clinical characteristics of dysglycemia and insulin resistance, vascular inflammation, blood pressure, and dyslipidemia. Statistics were maximized by assessing correlation analysis throughout the intervention study, including data from the 4 study visits for clinical assessment. For all subjects throughout the study, there were negative correlations of PBMC Glo1 activity with plasma protein MG-H1 and plasma D-lactate, suggesting that the increase of Glo1 may suppress both protein glycation by MG and, after improvement in metabolic health, flux of formation of MG as well. For clinical variables, BMI and AUCg correlated positively with plasma D-lactate, and the OGIS index correlated negatively with plasma D-lactate. This suggests that flux of MG is positively associated with dysglycemia, insulin resistance, and BMI.

For vascular inflammation markers, plasma MCP-1, sVCAM1, and sICAM1 correlated negatively with PBMC Glo1 activity and plasma soluble E-selectin (sE-selectin) correlated positively with plasma D-lactate. For blood pressure, systolic and diastolic blood pressure correlated positively with plasma MG concentration. Diastolic blood pressure and plasma endothelin-1 (ET-1) correlated negatively to PBMC Glo1 activity and positively with plasma D-lactate. For variables of clinical dyslipidemia, HDL correlated negatively and LDL-VLDL and TG positively with urinary MG-H1—a measure of total body MG glycation [15]; and plasma D-lactate correlated positively with TC, LDL-VLDL, and TG and negatively with HDL. This suggests increasing Glo1 expression may have health benefits through decreasing vascular inflammation and improving hemodynamics and dyslipidemia (Table 1). There was also a strong positive correlation of total urinary metabolites of tRES and total urinary metabolites of HESP (r = 0.84, p = 2 × 10^−7^).

### 3.2. Correlation Analysis of Changes from Baseline of Glyoxalase Pathway and Clinical Variables in tRES-HESP and Placebo Treatment Periods

To assess clinical variables linked to pharmacological responses of tRES-HESP, we performed correlation analysis of glyoxalase pathway and clinical variables significantly changed from baseline in tRES-HESP treatment period, comparing these changes with placebo. In the tRES-HESP treatment period only, change in plasma MG correlated negatively with change in FMD-GTN and change in PBMC quinone reductase (NQO1) activity in highly overweight and obese subjects. This suggests decrease of plasma MG is associated with improved arterial dilatation response and increased NQO1 activity—a marker of Nrf2 activation [22]. For dysglycemia/insulin resistance linked variables, there were negative correlations of OGIS index with FPG, AUCg, and plasma insulin OGTT in all and the highly overweight and obese subject group. For change in AUCg, there was a positive correlation with change in sE-selectin in all subjects. In highly overweight and obese subjects, there was also a positive correlation of OGIS with urinary pentosidine. There was also a negative correlation of change in FPG with change in PBMC NQO1 in all subjects; and in highly overweight and obese subjects, there was a negative correlation of change in FPG with change in urinary pentosidine. 

Several correlations between OGIS index, AUCg, and plasma insulin OGTT were found in both tRES-HESP treatment and placebo periods, as expected for the inverse relationship of insulin sensitivity to dysglycemia and hyperinsulinemia in subjects independent of treatment (Table 2).

### 3.3. Correlation Analysis of PBMC Gene Expression Changed in tRES-HESP and Placebo Treatment Periods

**Figure 2:**
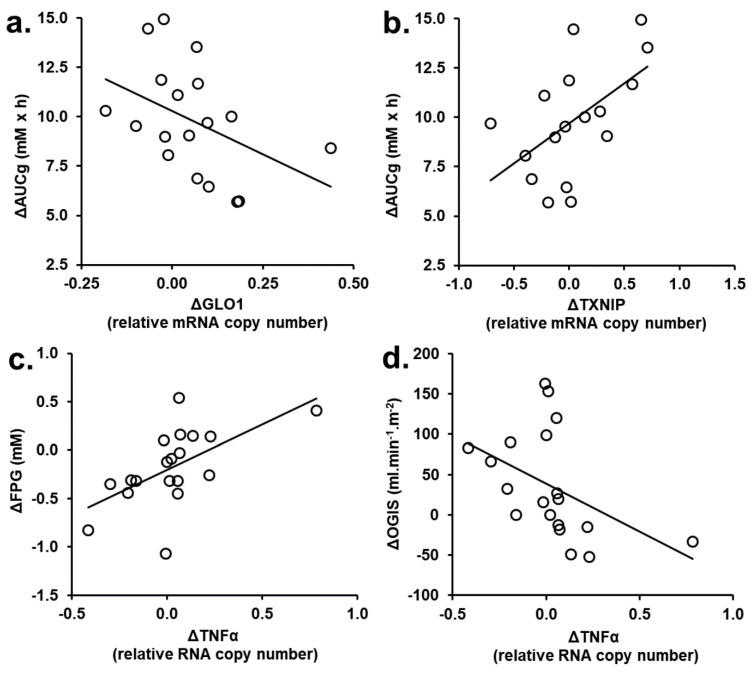
Correlation of variables of metabolic health with change in PBMC gene expression in highly overweight and obese subjects of the HATFF study during treatment with tRES-HESP. (**a**) Change of AUCg (ΔAUCg) on change expression of Glo1 (ΔGLO1); r = −0.56, *p* < 0.05. Regression equation: ΔAUCg = (−8.8 × ΔGLO1) + 10.3. (**b**) Change of AUCg (ΔAUCg) on change in expression of TXNIP (ΔTXNIP); r = 0.59, *p* < 0.05. Regression equation: ΔAUCg = (4.1 × ΔTXNIP) + 9.7. (**c**) Change in FPG (ΔFPG) on change in expression of TNFα (ΔTNFα); r = 0.70, *p* < 0.001. Regression equation: ΔFPG = (0.94 × ΔTNFα) − 0.20. (**d**) Change in OGIS (ΔOGIS) on ΔTNFα; r = −0.68, *p* < 0.01. Regression equation: ΔOGIS = (−119 × ΔTNFα) + 39. *n* = 20.

To assess PBMC gene expression changes linked to pharmacological responses of tRES-HESP, we performed correlation analysis of genes with expression changed from baseline in tRES-HESP treatment period with other gene expression assessed in the Nanostring expression array, comparing these to the placebo. In the tRES-HESP treatment period, most significant changes in PBMC gene expression were found in the highly overweight and obese subjects. Exploring the correlation of change in PBMC gene expression with clinical and metabolic variables, we found change in Glo1 expression correlated negatively with change in AUGg (r = −0.56, *p* < 0.05) and change in thioredoxin interacting protein (TXNIP) correlated positively with change in AUGg (r = 0.59, *p* < 0.05); and change in expression of tumor necrosis factor-α (TNFα) correlated positively with change in FPG (r = 0.70, *p* < 0.001) and negatively with change in OGIS (r = −0.68, *p* < 0.01). These correlations were not present with the placebo (Figure 2).

Major changes in gene expression in the tRES-HESP treatment period were: CCL2, −49%; IL-8, −39%; COX-2, −31%; and RAGE, −37%—as reported previously [15]. Change in expression of COX-2 correlated positively with change in expression of IL-8. Change in expression of CCL2, IL-8, and RAGE were intercorrelated and also all correlated with change in expression of the following genes: positive correlation—Mondo A (MLXIP), small maf protein, isoforms F and G (MAFF and MAFG), neutrophil cytosol factor-1 (NCF1, p47phox), and ferritin (FTH1); and negative correlation—heme oxygenase-1 (HMOX1) and transketolase (TKT). Change in expression of CCL2 correlated positively with change in expression of a further 11 genes: aldoketo reductase 1C1 (AKR1C1), glucose-6-phosphate dehydrogenase (G6PD), γ-glutamylcysteine ligase-modulatory subunit (GCLM), glutathione peroxidases, isoforms 1 and 4 (GPX1 and GPX4), glutathione reductase (GSR), interleukin-6 (IL-6), transcription factor Nrf2 (NFE2L2), NF-κB inhibitor-alpha (NFKBIA), NQO1 and superoxide dismutase-1 (SOD1); and correlated negatively with glutathione S-transferase P1 (GSTP1). Change in expression of IL-8 correlated positively with change in expression of a further 3 genes: AKR1C1, NQO1, and SOD1. Change in expression of RAGE correlated positively with change in expression of a further 7 genes: catalase (CAT), G6PD, GCLM, GPX4, Kelch-like ECH-associated protein 1 (KEAP1), NFKBIA, and SOD1; and correlated negatively with change in expression of CCL2 receptor (CCR2). Several of these correlations were found in the placebo arm of the study (Table 3).

## 4. Discussions

Reversal of insulin resistance in overweight and obese subjects with tRES-HESP treatment in the HATFF study was associated with decreased dysglycemia, blood pressure, vascular inflammation, and dyslipidemia—a remarkable multiplicity of pathogenic processes. Moreover, health improvement was linked to two established mediators of insulin resistance: TXNIP and TNFα. TXNIP is a mediator of insulin resistance in liver, skeletal muscle and adipose tissue, and impaired pancreatic beta-cell insulin secretion [23,24,25] and TNFα decreases insulin receptor signaling in adipose tissue and skeletal muscle, particularly prior to development of T2DM [26,27,28]. tRES-HESP treatment produced a decrease of PBMC TNFα expression in the obese subject subgroup of the HATFF study [15]. Reversal of insulin resistance by tRES-HESP was not achieved by tRES and HESP individually in clinical evaluation [3,29], suggesting pharmacological synergism of tRES-HESP. The dose of HESP given was likely sufficient to inhibit intestinal glucuronosyl transferase and facilitate uptake of unconjugated tRES and HESP [6]. The outcomes of the HATFF study may provide a pointer to effective synergistic combinations of dietary polyphenols to counter insulin resistance.

In consideration of the correlation analysis of glyoxalase pathway and clinical variables throughout the HATFF study, increased Glo1 activity decreases the cellular and extracellular concentration of MG in *in vitro* studies [9,14]. Hence, increased Glo1 expression in PBMCs and other cells in response to tRES-HESP treatment *in vivo* is expected to decrease plasma MG and plasma protein glycation by MG. Consistent with this, we found a negative correlation of PBMC Glo1 activity to plasma protein MG-H1. PBMC Glo1 activity also correlated negatively with markers of vascular inflammation and blood pressure. A hypertensive effect of increased MG glycation may be mediated through activation of the UPR by MG-modified proteins and associated inflammatory signaling through the heat shock factor-1 and NF-κB pathways, with increased expression and secretion of ET-1 [9,30]. Hypertension in obesity was linked to enhanced vascular activity of endogenous ET-1 [31]. tRES decreased ET-1 expression in endothelial cells *in vitro* [32]. Increased peripheral resistance to blood flow may also contribute to a hypertensive response. The negative correlation of change in plasma MG with FMD-GTN, an indicator of nitric oxide-independent arterial dilatation, suggests increased MG may support this. Indeed, plasma MG was an independent risk factor of increased arterial intimal-medial thickness, pulse-wave velocity and systolic blood pressure in patients with T2DM [33]. Urinary excretion of MG-H1 correlated positively with LDL-VLDL and TG and negatively with HDL. Urinary flux of MG-H1 reflects total body flux of protein glycation by MG, with a contribution from MG-H1 free adduct absorbed from digested glycated proteins in food [15,34]. These associations are consistent with formation of pro-atherogenic small, dense TG-rich LDL by MG modification of LDL, and decreased stability and half-life of HDL when modified by MG [35,36]. 

For changes from the baseline of clinical variables in the tRES-HESP treatment period, the positive correlation of change in the OGIS index with change in urinary pentosidine may reflect formation of pentosidine as a marker of pentosephosphate pathway metabolism [37]. Induction of the ARE-linked gene, G6PD, by tRES-HESP increases the flux through the pentosephosphate pathway and may thereby increase formation of pentose precursors of pentosidine; this also decreases glucose-6-phosphate-dependent carbohydrate response element/Mondo A (MLXIP) transcriptional response [9], which may contribute to reversal of insulin resistance [38]. 

For changes in gene expression of PBMCs, there was a strong anti-inflammatory response induced by tRES-HESP—characterized by decreased expression of COX-2, MCP-1, IL-8, and RAGE [15]. This anti-inflammatory effect has not been achieved in clinical studies with tRES alone: for example, 150 mg tRES daily for 4 weeks in obese men had no effect on IL-8 [39] and 500 mg tRES daily for 4 weeks in obese men had no effect on MCP-1 [40]. Change in expression of MCP-1, IL-8 and RAGE correlated strongly with change in expression of Mondo A. Increased expression of Mondo A in skeletal muscle is linked to lipid accumulation and insulin resistance [38]. A similar positive correlation with FTH1 and a negative correlation with HMOX1 suggested there may be decreased availability of iron and increased metabolism of heme iron during treatment with tRES-HESP. Iron metabolism has an important role in immunity [41], increasing inflammatory activity of macrophages and correlating with BMI increase in the healthy population [42]. The positive correlation with expression of NCF-1 of the NADPH oxidase system may relate to its regulation by MCP-1 and priming of NADPH oxidase by IL-8 [43], contributing to systemic oxidative stress in clinical obesity [44]. Several genes with positive correlations to MCP-1, IL-8, and RAGE—AKR1C1, G6PD, GCLM, GPX1, GPX4, GSR, MAFFF, MAFG, NFE2L2, and NQO1 are cytoprotective genes regulated by Nrf2—including autoregulation of Nrf2 itself [45]. This may reflect a host counter-response to low-grade inflammation sustained by these inflammatory mediators.

Decrease of MCP-1 and IL-8 by tRES-HESP may be a downstream effect of improved insulin sensitivity rather than an upstream mediator of it. Plasma MCP-1 concentration was not associated with insulin resistance clinically and overexpression of MCP-1 in mice induced inflammation without insulin resistance or dysglycemia [46,47]. However, adipose tissue levels of MCP-1 and IL-8 were increased by hyperinsulinemic clamp [48]. If the anti-inflammatory effects of tRES-HESP on gene expression in PBMCs translate to tissues, there may be additional health benefits through a decrease of insulin resistance-associated low-grade inflammation in development of non-alcoholic fatty liver disease [49,50,51], chronic kidney disease [52,53], decline of respiratory function [54,55,56], cardiovascular disease, and aging [57,58,59]. COX-2 may be decreased through its regulation by MCP-1 [60]; and decreased expression and *in situ* activity of RAGE through decrease of MG-H1 which is a ligand for RAGE [61].

**Figure 3:**
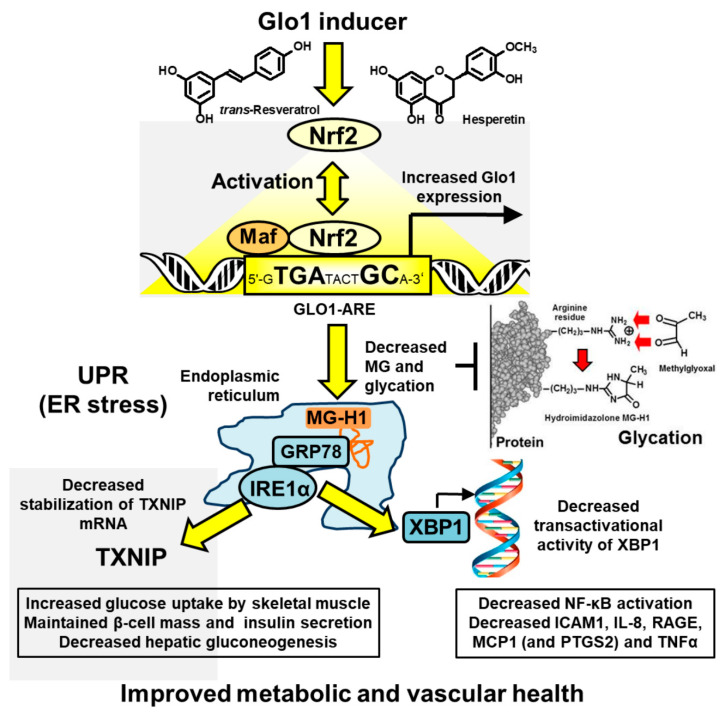
Proposed mechanism of action of Glo1 inducer, tRES-HESP, through suppression of the unfolded protein response. Key: yellow filled arrows—mechanism of health improvement; red filled arrows—damaging processes suppressed. See text for details.

Upstream signaling of MCP-1, IL-8, RAGE, and COX-2 may be linked to activation of the UPR by increased MG-modified misfolded proteins [9,10] (Figure 3). In UPR activation, IRE1α stabilizes TXNIP mRNA to increase its expression and activity [62]. TXNIP decreases glucose uptake by skeletal muscle and pancreatic beta-cell mass and insulin secretion and increases hepatic gluconeogenesis [23,63,64]. Inflammatory signaling may be mediated through X box-binding protein 1 (XBP1), increasing histone H3 lysine 4 methyltransferase, SET7/9, increasing expression of p65 of the NF-κB system and inflammatory mediators [65,66], including TNFα as key contributor to insulin resistance in skeletal muscle [13,67]. Treatment with tRES-HESP alleviates these UPR-mediated responses [9,10]; tRES-HESP may also decrease expression of TXNIP directly via Nrf2 and ARE-linked suppression [68]. tRES-HESP may thereby be a potent inducer of reversal of insulin resistance with applications for both prevention and treatment of T2DM.

Interestingly, since conducting the HATFF study, studies with human aortal endothelial cells and periodontal ligand fibroblasts in primary culture and model hyperglycemia have indicated that tRES-HESP may correct endothelial dysfunction and periodontal ligament dysfunction associated with hyperglycemia in prediabetes and diabetes [9,10]. tRES-HESP also corrected dysfunction of bone marrow progenitor cell in experimental diabetes, improving wound closure and angiogenesis in diabetic mice [69]. Suppression of activation of the UPR was implicated in these responses.

In conclusion, correlation analysis of data from the HATFF study indicates that the responses of optimised Glo1 inducer, tRES-HESP, are linked to improvements in dysglycemia, blood pressure, dyslipidemia, and low-grade inflammation. The reversal of insulin resistance induced by tRES-HESP was related inversely to expression of TNFα and TXNIP and may reflect countering of MG accumulation and protein glycation, with consequent decreased activation of the UPR [70].

## Figures and Tables

**Table 1:** Correlation of variables throughout the HATFF study in all subjects.

Variable Class	Correlate	PBMC Glo1	MG_Plasma_	MG-H1_urine_	D-Lactate_Plasma_
Glyoxalase pathway	Plasma protein MG-H1	−0.21 *			
	Plasma D-lactate	−0.34 ***			
Anthropometric	BMI				0.38 ***
Dysglycemia/ insulin resistance	AUCg				0.25 **
	OGIS				−0.23 *
Vascular inflammation	Plasma MCP-1	−0.28 **			
	Plasma sVCAM1	−0.18 *			
	Plasma sICAM1	−0.22 *			
	Plasma sE-selectin				0.24 **
Blood pressure	Systolic BP		0.26 **		
	Diastolic BP	−0.24 **	0.23 *		0.23 *
	Plasma ET-1	−0.24 **			0.25 **
Dyslipidemia	TC				0.19 *
	HDL			−0.20 *	−0.19 *
	LDL-VLDL			0.39 ***	0.26 **
	TG			0.28*	0.20 *

Spearman correlation coefficients; *n* = 116. Significance: *, *p* < 0.05; **, *p* < 0.01, and ***, *p* < 0.001. Estimates of variables at all four study visits and both tRES-HESP and placebo treatments were included in the analysis.

**Table 2:** Correlation of changes of glyoxalase and dysglycemia/insulin resistance-related variables in the tRES-HESP and placebo treatment periods of the HATFF study.

Variable Class	Variable	Study Group	Correlate
Glyoxalase pathway	Plasma MG	Highly overweight and obese	FMD-GTN (−0.48 *), PBMC NQO1 (−0.68 **)
Dysglycemia/insulin resistance	OGIS index	All	FPG (−0.79 ***), AUCg (−0.50 **), plasma insulin OGTT (−0.63 ***)
		Highly overweight and obese	FPG (−0.80 ***), AUCg (−0.57 **), plasma insulin OGTT (−0.57 **), urinary pentosidine (0.54 *)
	FPG	All	OGIS index (−0.79 ***), PBMC NQO1 (−0.50 *)
		Highly overweight and obese	OGIS index (−0.80 ***), urinary pentosidine (−0.56 *)
	AUCg	All	OGIS (−0.46 **), sE-selectin (0.47 *)
		Highly overweight and obese	OGIS (−0.57 **)

Spearman correlation coefficients; *n* = 20 (highly overweight and obese) and *n* = 29 (all). Significance: *, *p* < 0.05; **, *p* < 0.01 and ***, *p* < 0.001. Underlined correlates indicate correlation is also found in both tRES-HESP and placebo treatment periods of the HATFF study.

**Table 3:** Correlation of changes of peripheral blood mononuclear gene expression in highly overweight and obese subjects in the tRES-HESP and placebo treatment periods of the HATFF study.

Gene	Gene Correlate (Correlation Coefficient)
COX-2	IL-8 (0.74 *)
CCL2	AKR1C1 (0.80 **), CCR2 (−0.83***), FTH1 (0.89 ***), G6PD (0.77 **), GCLM (0.93 ***), GPX1 (0.77 **), GPX4 (0.85 ***), GSR (0.70 *), GSTP1 (−0.69 *), HMOX1 (−0.88 ***), IL-6 (0.79 **), IL-8 (0.74 *), MAFF (0.76 **), MAFG (0.81 **), MLXIP (0.92 ***), NCF1 (0.74 *), NFE2L2 (0.75 *), NFKBIA (0.70 *), NQO1 (0.79 **), RAGE (0.87 ***), SOD1 (0.86 ***), TKT (−0.88 ***).
IL-8	AKR1C1 (0.73 *), CCL2 (0.74 *), COX-2 (0.74 *), FTH1 (0.88 ***), MAFG (0.71 *), MLXIP (0.69 *), NCF1 (0.72 *), NQO1 (0.76 **), RAGE (0.70 *), SOD1 (0.73 *).
RAGE	CAT (−0.71 *), CCL2 (0.87 *), CCR2 (−0.74 *), FTH1 (0.84 ***), G6PD (0.86 ***), GCLM (0.76 **), GPX4 (0.71*), HMOX1 (−0.80 **), IL-8 (0.70 *), KEAP1 (0.69 *), MAFF (0.83 ***), MAFG (0.91 ***), NFKBIA (0.75 **), MLXIP (0.92 ***), NCF1 (0.88 ***), SOD1 (0.86 ***), TKT (−0.81 **).

Spearman correlation coefficients; *n* = 20. Significance: * *p* < 0.05; ** *p* < 0.01; and *** *p* < 0.001 (Bonferroni correction of 47 applied). Gene name correlates underlined had changes correlated similarly in the placebo arm. Gene expression assessed was: AGER, AKR1B1, AKR1C1, AKR1C3, CAT, CBR1, CCL2, CCR2, CD36, FTH1, G6PD, GCLC, GCLM, GPX1, GPX4, GSR, GSTA4, GSTP1, HIF1A, HMOX1, IL-6, IL-8, KEAP1, MAFF, MAFG, MAFK, MIF, MLX, MLXIP, NCF1, NFE2L2, NFKB1, NFKBIA, NQO1, PRDX1, PSMA1, PSMB5, PTGS2, SOD1, SQSTM1, SREBF1, TALDO1, TKT, TNFA, TXN, TXNIP, TXNRD1. Housekeeping reference genes: ACTB, CLTC, and GUSB.

## Data Availability

The data presented in this study are available on request from the corresponding author.
